# A70 FECAL-ADHERENT MUCUS IS A NON-INVASIVE SOURCE OF PRIMARY HUMAN MUC2 FOR STRUCTURAL AND FUNCTIONAL CHARACTERIZATION IN HEALTH AND DISEASE

**DOI:** 10.1093/jcag/gwac036.070

**Published:** 2023-03-07

**Authors:** N Fancy, M Melvin, N N, N Kazemian, L D’Aloisio, S Davidson-Hunt, K Chadee, S Pakpour, S Ghosh, D Gibson, W Zandberg, K Bergstrom

**Affiliations:** 1 Biology; 2 Chemistry; 3 School of Engineering, University of British Columbia - Okanagan, Kelowna; 4 Cumming School of Medicine, University of Calgary, Calgary, Canada

## Abstract

**Background:**

The gel-forming O-glycoprotein Mucin-2 (MUC2) is a key mediator of host-microbe homeostasis in part by forming a barrier to segregate inflammatory microbes from distal colon tissues. While animal models have provided insights into how Muc2 functions, relatively few studies have explored human MUC2. This is due to difficulty in accessing primary MUC2 which has been traditionally seen as firmly adherent to tissues and thus attainable mainly through invasive approaches (e.g. surgery, biopsies, etc), or via transformed cell lines (e.g. LS174T). This highlights a need to find alternative sources of MUC2.

**Purpose:**

The purpose of this study is to develop a non-invasive method to analyze human MUC2 from healthy persons and determine if this can be applied to disease states. Recent studies have shown a significant portion of MUC2 is bound to feces (Bergstrom and Shan et al, 2020). We therefore reasoned this fecal MUC2 was accessible for both purification and structural and functional characterization.

**Method:**

We purified MUC2 from feces via established extraction methods used for tissues. The mucins were resolved by composite urea agarose polyacrylamide gel electrophoresis (UreaAgPAGE) and analyzed by in-gel staining with Alcian Blue (AB), or Western blot for lectins and MUC2. Mucins were subjected to both proteomics to confirm enrichment of MUC2; and O-glycomics via non-reductive ammonia-catalyzed β-elimination followed by capillary electrophoresis (CE) and mass spectrometry in parallel with Type III porcine gastric mucin (PGM) O-glycans for comparison. Purified O-glycans were tested functionally via microbial growth assays with *Bacteroides* spp, and the ability to be metabolized into short-chain fatty acids (SCFA). Mucus barrier function was also visualized directly on Carnoy's-fixed paraffin-embedded (CFPE) fecal sections followed by dual staining for bacteria by FISH, and MUC2 and/or lectins.

**Result(s):**

Confocal imaging of CFPE fecal sections revealed a microbiota-encapsulating barrier layer of varying thickness among various healthy human subjects. UreaAgPAGE showed high molecular weight bands (~1 – 2 MDa) by AB staining. Proteomics and western analysis confirmed MUC2 enrichment in fecal mucin preparations. Western analysis via a lectin panel showed human MUC2 bound several lectins but was notably lacking in signal for *Sambucus nigra* lectin (SNA; α2,6-linked sialic acid) or *Ulex europaeus agglutinin I* (UEA1; α1,2-linked fucose). O-glycomics revealed extensive sialylation, moderate sulfation, and very little fucosylation vs. PGM. Functionally, the glycans supported growth of *Bacteroides thetaiotaomicron* as well as *B.theta*-dependent SCFA production in vitro. Pilot studies with human Ulcerative Colitis (UC) showed intact MUC2 with a differential O-glycosylation profile by glycan "fingerprinting" via CE.

**Conclusion(s):**

These studies highlight a new way to access primary human MUC2 for downstream functional analyses and pave the way for characterizing MUC2 dysfunction in diseases including IBD.

**Disclosure of Interest:**

None Declared

A71

**SIGNIFICANT RACIAL/ETHNIC DIFFERENCES EXIST IN THE RECEIPT OF IBD-RELATED SURGERY:**

**A SYSTEMATIC REVIEW AND META-ANALYSIS**

T. Chhibba^*^, P. Tandon, N. Natt, G. Brar, G. Malhi, G. Nguyen

**Background:**

Patients with inflammatory bowel disease (IBD) may require surgical intervention for management of their disease. There is a rising incidence of IBD in racial and ethnic minorities but studies regarding healthcare utilization patterns in these populations have yielded variable results.

**Purpose:**

We aimed to examine the differences in surgical rates of ethnic and racial groups compared to White patients with IBD.

**Method:**

Electronic databases were searched through December 20, 2021. Studies that compared ulcerative colitis (UC) or Crohn’s disease (CD) surgery rates between different racial/ethnic groups were included. Both pediatric and adult studies were included. Pooled event rates were generated and p-value < 0.05 was considered statistically significant in generating odds ratios (OR) with 95% confidence interval (CI). We also compared differences in disease location, phenotype, and IBD-medication exposure amongst different groups included.

**Result(s):**

Forty-one studies stratified rates of IBD-related surgeries by race or ethnicity (n=1,094,693 patients). Black patients were less likely to undergo IBD-related surgeries compared to White patients (pooled OR 0.70, 95% CI, 0.55-0.89, I2=87.0%). Black patients were also less likely compared to White patients to undergo an emergent colectomy with an incidence rate ratio of 0.43 (95% CI, 0.32-0.58). Furthermore, Hispanic patients were less likely to undergo a CD-related surgery (pooled OR 0.57, 95% CI, 0.48-0.68, I2=0%) compared to White patients. Finally, Asian patients had no significant difference in likelihood of CD-related and UC-related surgeries compared to White patients. Black patients were more likely to have perianal disease (pooled OR 1.40, 95% CI, 1.06-1.86), I2=58.2%) but otherwise disease characteristics and phenotypes were similar across all populations compared to Caucasians.

**Conclusion(s):**

Black and Hispanic patients with IBD are less likely to have surgery, including emergent surgery, for IBD compared to White patients with IBD, despite similar disease phenotype characteristics. Disparities in access to care may be contributory toward these findings and efforts should be made to provide equitable care to all persons living with IBD, regardless of race and ethnicity.

**Please acknowledge all funding agencies by checking the applicable boxes below:**

Other

**Please indicate your source of funding below:**

Nil

**Disclosure of Interest:**

None Declared

